# Cholesterol Influences Voltage-Gated Calcium Channels and BK-Type Potassium Channels in Auditory Hair Cells

**DOI:** 10.1371/journal.pone.0026289

**Published:** 2011-10-14

**Authors:** Erin K. Purcell, Liqian Liu, Paul V. Thomas, R. Keith Duncan

**Affiliations:** Department of Otolaryngology, University of Michigan, Ann Arbor, Michigan, United States of America; University of Houston, United States of America

## Abstract

The influence of membrane cholesterol content on a variety of ion channel conductances in numerous cell models has been shown, but studies exploring its role in auditory hair cell physiology are scarce. Recent evidence shows that cholesterol depletion affects outer hair cell electromotility and the voltage-gated potassium currents underlying tall hair cell development, but the effects of cholesterol on the major ionic currents governing auditory hair cell excitabilityare unknown. We investigated the effects of a cholesterol-depleting agent (methyl beta cyclodextrin, MβCD) on ion channels necessary for the early stages of sound processing. Large-conductance BK-type potassium channels underlie temporal processing and open in a voltage- and calcium-dependent manner. Voltage-gated calcium channels (VGCCs) are responsible for calcium-dependent exocytosis and synaptic transmission to the auditory nerve. Our results demonstrate that cholesterol depletion reduced peak steady-state calcium-sensitive (BK-type) potassiumcurrent by 50% in chick cochlear hair cells. In contrast, MβCD treatment increased peak inward calcium current (∼30%), ruling out loss of calcium channel expression or function as a cause of reduced calcium-sensitive outward current. Changes in maximal conductance indicated a direct impact of cholesterol on channel number or unitary conductance. Immunoblotting following sucrose-gradient ultracentrifugation revealed BK expression in cholesterol-enriched microdomains. Both direct impacts of cholesterol on channel biophysics, as well as channel localization in the membrane, may contribute to the influence of cholesterol on hair cell physiology. Our results reveal a new role for cholesterol in the regulation of auditory calcium and calcium-activated potassium channels and add to the growing evidence that cholesterol is a key determinant in auditory physiology.

## Introduction

Cholesterol is an integral component of the cell membrane and regulates the activity of ion channels in the lipid bilayer. Potential mechanisms of influence include: (1) direct interaction with the channel protein, (2) changes in the fluidity of the bilayer which affect ion channel gating and conformational change, or (3) compartmentalization of ion channels into spatially restricted signaling complexes (“lipid rafts”) [1], [2]. The concept of lipid rafts emerged in the study of intestinal epithelial cells, where a polarized distribution of membrane lipids accompanies segregated trafficking at the apical and basolateral sides of the cell [3], [4]. Hair cells in the inner ear are uniquely asymmetric cells in both form and function, with a mechanotransduction complex composed of stereocilia embedded in a rigid cuticular plate located at the apical end and the intricate machinery of synaptic transmission clustered at the basolateral pole. While the finely tuned interplay of the ionic currents responsible for transducing the intensity, temporal, and frequency characteristics of sound to the brain is widely appreciated [5],[6], the role of the local lipid environment in coordinating ion channel physiology in auditory hair cells is largely unexplored.

Four major ion channel subtypes contribute to the membrane potential of the afferently innervated tall hair cells of the chick cochlea: a calcium-activated potassium channel (BK), a voltage-gated calcium channel (VGCC), an inwardly rectifying potassium channel (Kir), and a slow delayed rectifier potassium channel (Kv) [7]. Each of these channel types plays an essential role in hearing, and all have shown sensitivity to membrane cholesterol content in various cellular models [2]. The large conductance, calcium-activated, ‘BK’-type potassium channels play a variety of roles in the physiological functions of numerous cellular systems. In inner ear hair cells, BK channels are responsible for the temporal precision of sound encoding in mammals [6]. In non-mammals, BK expression and kinetics are responsible for setting the resonant frequency of afferently-innervated hair cells [8]. BK channels shape the receptor potential generated by inner hair cells, as evidenced by slowed voltage responses of inner hair cells in mice lacking the pore-forming alpha subunit of BK [6]. The importance of BK channels in audition is underpinned by their expression at the onset of hearing (∼E18 in chicks and ∼P12 in mouse) [9], [10]. While cholesterol reportedly modulates BK currents in smooth muscle, glioma, neuronal and endothelial cells [2], [11], [12], a functional role in auditory hair cells has not been reported previously.

In non-mammals, L-type voltage-gated calcium channels serve as the calcium source for BK channels. In all vertebrates, L-type VGCCs also play a key role in neural transmission from the hair cell to the auditory nerve, triggering exocytosis at the basolateral end of the hair cell [13]. Due to high calcium buffering, VGCCs must be in close proximity to BK and to the calcium sensors driving vesicular fusion [14]. Cholesterol inhibits L-type VGCCs in cardiac and coronary myocytes, where cholesterol chelation with MβCD enhances channel activity [15], [16]. However, similarly to the BK channel, the role of cholesterol in modulating VGCCs varies depending upon the particular preparation and cell type studied.

Kv and Kir channels counteract depolarization and hyperpolarization of the cell membrane respectively. Kv and Kir channels underlie tuning in the low frequency, apical hair cells of non-mammals [17]. Kv currents play a specialized role in hair cell development, repolarizing the spontaneous action potentials (SAPs) credited with directing the tonotopic organization of the auditory periphery [18], [19]. Cholesterol depletion with MβCD potentiates Kv currents in developing auditory hair cells and abolishes SAPs [20]. Kir currents display sensitivity to cholesterol depletion in a variety of cell types, but the role of cholesterol in modulating Kir in auditory hair cells is unknown[2].

We investigated the effects of the cholesterol depleting drug, MβCD, on the macroscopic currents of the four major ion channel classes responsible for determining the membrane potential of chick auditory hair cells. Depleting cholesterol from the hair cell membrane reduced BK currents while VGCC conductance was increased. There were lesser or absent effects of cholesterol depletion on Kv and Kir currents respectively. Cholesterol staining was most intense at the apical and basolateral ends of the cell and BK channels were identified in cholesterol-enriched microdomains. Our data show that the lipid environment modulates ion channels essential for sound processing and synaptic transmission in the auditory hair cell membrane.

## Methods

### Ethics statement

The care, maintenance, and treatment of animals in these studies followed protocols approved by the University Committee on Care and Use of Animals at the University of Michigan (protocol #08824-3). Chicks were deeply anesthetized with a ketamine/xylazine cocktail and euthanized prior to tissue harvest.

### Hair cell dissociation

Hair cells were harvested from post-hatch chicks (*Gallus gallus*), ranging from 5 to 19 days old as previously described [21]. Cells were derived from a total of nine chicks for I_K(Ca)_recordings, and nineteen chicks were used to obtain cells for calcium traces. The basilar papilla was extracted through the oval window and exposed to artificial perilymph (154 mMNaCl, 6 mMKCl, 5 mM CaCl_2_, 2 mM MgCl_2_, 5 mM HEPES, 8 mM glucose buffered to pH 7.4 with NaOH) supplemented with 0.01 % protease (Type XXIV) for 1 minute. The basilar papilla was microdissected to expose the sensory epithelium, and the segment measuring 1.2–2.0 mm from the apical end was isolated. This region is known to contain BK-expressing afferently-innervated tall hair cells [22], [23], which are analogous in many ways to the inner hair cells of the mammalian cochlea. Individual hair cells were dissociated by aspirating the segment through a fine-tipped borosilicate glass pipette. Cells were then exposed for 20 minutes to either (1) control artificial perilymph or (2) 1 mM MβCD dissolved in artificial perilymph. The solution was aspirated and replaced by fresh artificial perilymph for patch clamp recordings.

### Filipin staining

Filipin stain (Sigma) was used to assess cholesterol distribution in the hair cell membrane in control and MβCD-treated cells [20]. Filipin was dissolved in DMSO to prepare a 50 mg/mL stock solution. Dissociated hair cells were fixed in 2% paraformaldehyde in phosphate buffer, rinsed and treated with a quenching solution (1.5 mg/mL glycine, 1% bovine serum albumin (BSA), 0.02% saponin in PBS). Cells were incubated in 0.5 mg/mL filipin (stock diluted in PBS) in the dark, rinsed and mounted with ProLong Gold (Molecular Probes). Images were obtained with a Leica DM LB fluorescence microscope with a cooled-CCD colour digital camera (MicroPublisher, Q Imaging) with the UV filter set. Care was taken to keep exposure times constant between samples and minimize bleaching. Staining intensity was measured with ImageJ software, where the average pixel intensity of the soma and the line profile intensity as a function of distance from the basolateral pole to the bottom edge of the cuticular plate were measured. The latter measurement was normalized to the total cell length for each cell and expressed as percent distance from the hair cell base.

### Hair cell electrophysiology

Whole-cell patch-clamp recordings were made with an Axopatch 200B amplifier, Digidata 1322A digitizer, and the pClamp 9.0 software suite (Axon Instruments, Foster City, CA). Data were sampled at 20 kHz and low-pass filtered at 5 kHz. Leak currents were subtracted off-line. Uncompensated series resistance was not corrected. All recordings were made at room temperature (22–25°C). Microelectrodes were pulled from borosilicate glass capillaries (World Precision Instruments, Sarasota, FL) with aresistance of 6.9±1.2 MΩ (mean±STD), and cells had an average membrane capacitance of 7.4 pF and an average access resistance of 17 MΩ. For potassium current recordings, the external (bath) solution was artificial perilymph, and the internal (pipette) solution contained 112 mMKCl, 2 mM MgCl_2_, 0.1 mM CaCl_2_, 11 mM EGTA, 10 mM HEPES, and 5 mM Na_2_ATP. To identify the calcium-sensitive component of this current, a calcium-free solution was flowed onto the cell by a manual exchange 5 times the volume of the bath. This solution replaced CaCl_2_ in artificial perilymph with an equimolar concentration of MgCl_2_. Replacement of calcium in this manner produced a leftward shift in the activation of outward current. This effect was attributed to differences in surface screening and liquid junction potential upon external solution exchange and was corrected prior to subtraction [24], [25]. Fast K^+^ current was measured 1.5 msec following the stimulus onset in the presence of calcium [26]. For calcium current recordings, KCl in the internal solution was replaced by CsCl (120 mM), and 20 mMtetraethylammonium-Cl (TEA-Cl) was added in both internal and external solutions to block K^+^ currents. Conductance was calculated from steady-state calcium currents and estimates of the reversal potential. Conductance-voltage curves were fit with a single-order Boltzmann function, *G/G*max  =  1/{1 + exp[(*V*_half_ – *V*)/S]}, where *V*_half_ is the half-activation voltage, *V* is the voltage command, *G*max is maximum conductance, and *S* is the Boltzmann slope. All chemicals used in the electrophysiology studies were purchased from Sigma. Averaged values are reported as means±standard error of the mean.

### Sucrose gradient ultracentrifugation and immunoblotting

Whole cochlear ducts were extracted directly through the oval window. Control cochleae were incubated in artificial perilymph for 20 minutes at room temperature before being flash frozen on dry ice. Some samples were treated with 10 mM MβCD in artificial perilymph for 20 to 60 minutes at room temperature before freezing. In comparison to the 1 mM concentration of MβCD used for dissociated hair cells, the higher concentration of MβCD was required for penetration into intact cochleae. For both treatment groups, sixteen cochleae were pooled into a single tube to obtain sufficient material for further processing.

Samples were thawed on ice in 2(N-Morpholino)-ethane sulfonic acid (MES)-buffered saline (MBS) (25 mM MES, 150 mMNaCl, pH 6.5) containing 1% Triton X-100 and protease inhibitor cocktail (Sigma). After homogenizationwith a motorized pestle, lysates were held on ice for 20 to 30 minutes before fractionation. The homogenate with 40% (w/v) sucrose was placed on the bottom of a 2.2 ml ultracentrifuge tube. This solution was overlaid with 900 µl of 30% sucrose and 900 µl of 5% sucrose in MBS for a discontinuous gradient. Membrane proteins were separated using an Optima Max-E Ultracentrifuge with a swinging bucket rotor (TLS-55) (Beckman-Coulter, Fullerton, CA). The samples were ultracentrifuged at 200,000 g for 24 hours at 4°C. Twelve equal volume fractions (183 µl each) were collected from the top. Aliquots (25 µl) were taken from each fraction, mixed with 2X Laemmli sample buffer (Bio-Rad) containing 5% 2-mercaptoethanol, separated by SDS-PAGE on a 4–15% polyacrylamide gel, and transferred to nitrocellulose membranes (Pierce Biotechnology, Rockford, IL) for Western blotting. Membranes were probed with primary antibodies to BK (1∶200, BD Transduction Laboratories, Rockville, MD) and caveolin (1∶2000, pan-anti-CAV, BD Transduction Laboratories, Rockville, MD) and then stripped and re-probed with a primary antibody to human transferrin receptor (1∶2000 anti-TfR, Zymed Laboratories, Carlsbad, CA). Secondary antibodies included goat anti-mouse or anti-rabbit conjugated to horseradish peroxidase (1∶4000-10,000, Pierce Biotechnology). Reactions were visualized with ChemiGlow West chemiluminescent substrate (Cell Biosciences, Santa Clara, CA).

### Statistics

A linear mixed-model ANOVA was used to assess the effect of treatment condition on voltage-gated currents while controlling for correlated observations within the same cell. The random effect was the individual cell assessed, while voltage and treatment condition were included in the model as fixed factors. The analysis utilized a Toeplitz correlation structure and significant effects were compared using a least significant difference test. Mixed-model ANOVA was similarly employed to assess the effects of relative apical-basal location on filipin staining intensity. A Student's t-test was used to compare filipin intensity, maximal conductance (Gmax), and Boltzmann fit parameters between treated and untreated cells where indicated. Statistical significance was defined at the p<0.05 level.

## Results

### Hair cell cholesterol is depleted by MβCD

**Figure 1:**
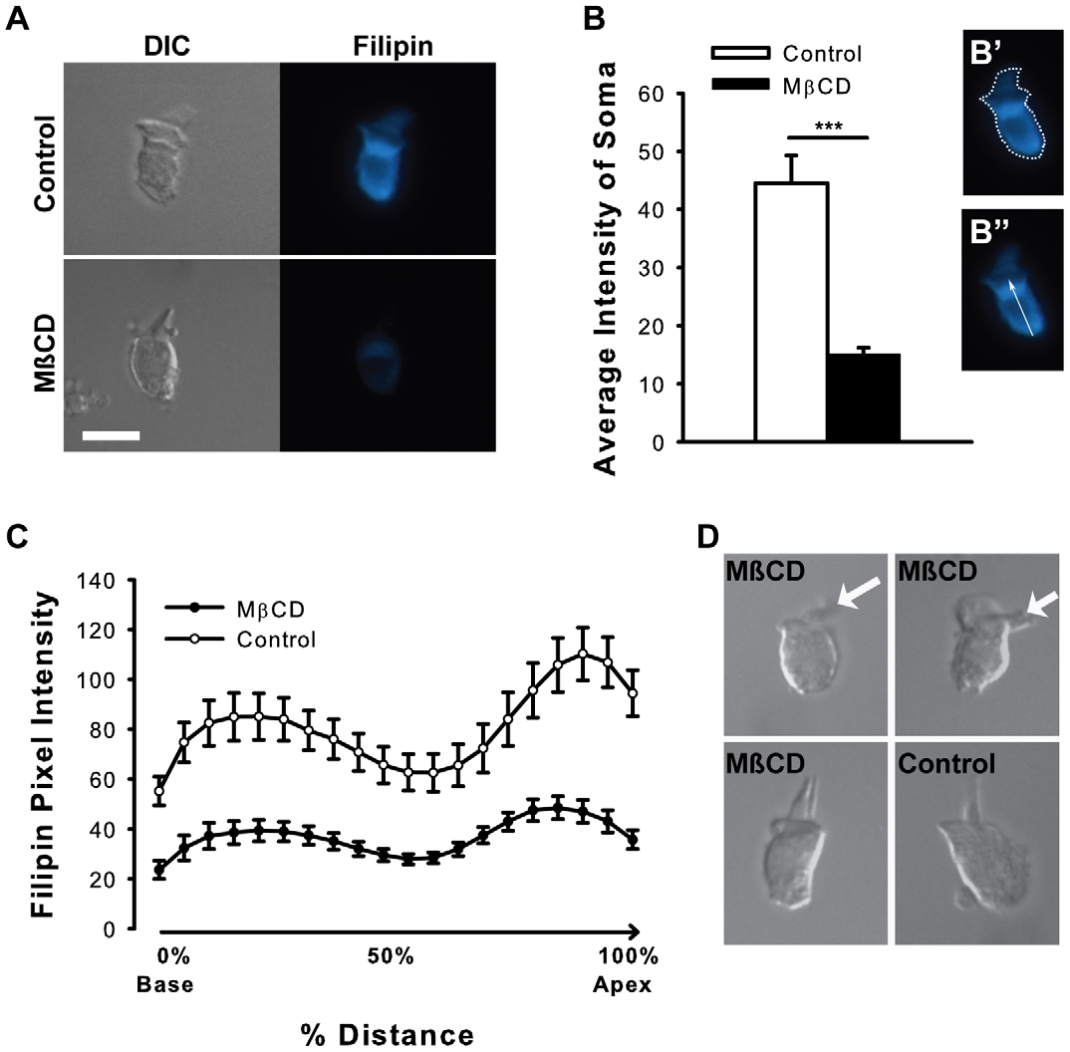
Hair cell cholesterol content is depleted by MβCD. (A) Representative images of filipin labeling (blue) show markedly reduced staining intensity in MβCD-treated cells. (B) Quantitative analysis shows a significant reduction in the average pixel intensity of the soma (region assessed depicted in B'). (C) Cholesterol staining peaks in the apical and basolateral ends of the cell, and MβCD reduces staining intensity by ∼50–60% while maintaining this distribution (line profile assessed depicted in B''). (D) MβCD-treatment showed an apparent loss of structural integrity at the bundle insertion at the cuticular plate (arrow highlights bundle orientation in top panels). The majority of MβCD-treated cells, as well as all untreated cells, had the expected perpendicular arrangement (bottom panels). *** = p< 0.001. Scale  =  10 microns.

We assessed the impact of MβCD on cellular cholesterol content by quantifying the intensity of filipin staining. While MβCD is the most efficient and most commonly used agent to deplete free cholesterol from the cell membrane, the cholesterol-extracting hydrophobic pocket of MβCD has the potential to interact with non-targeted membrane lipids and proteins [27]. Filipin labeling demonstrated pronounced extraction of cholesterol from auditory hair cells by MβCD (Figure 1A–C). There was a significant reduction in the average pixel intensity of somatic filipin labeling following MβCD treatment (Figure 1B-B') (p<0.001, n = 15 control and 27 MβCD cells, t-test). Line profiles of the staining intensity from the base to the apex of the cell revealedintensity peaks in the opposing ends of the cell (Figure 1C, see B'' for illustration of line profile). MβCD consistently reduced the filipin intensity along the axis of the cell by ∼50–60% regardless of the position along the length of the cell; cholesterol extraction was not preferential to a specific cellular region (Figure 1C) (p<0.001, n = 15 control and 27MβCD cells, ANOVA). Significant elevations in staining were noted in the regions 15–35% and 75–100% from the hair cell base, which may reflect the position of the nucleus and/or a polarized distribution of cholesterol in the membrane (Figure 1C) (p<0.05, n = 15 control and 27 MβCD cells, ANOVA). In some MβCD-treated cells, the hair bundle pivoted *en masse* relative to the cuticular plate (arrows, top panels of Figure 1D), losing the normal perpendicular arrangement observed in cells from both conditions (bottom panels, Figure 1D) while apparently retaining lateral rigidity between adjacent stereocilia.

### Cholesterol depletion with MβCD reduces BK-type outward currents in auditory hair cells

**Figure 2:**
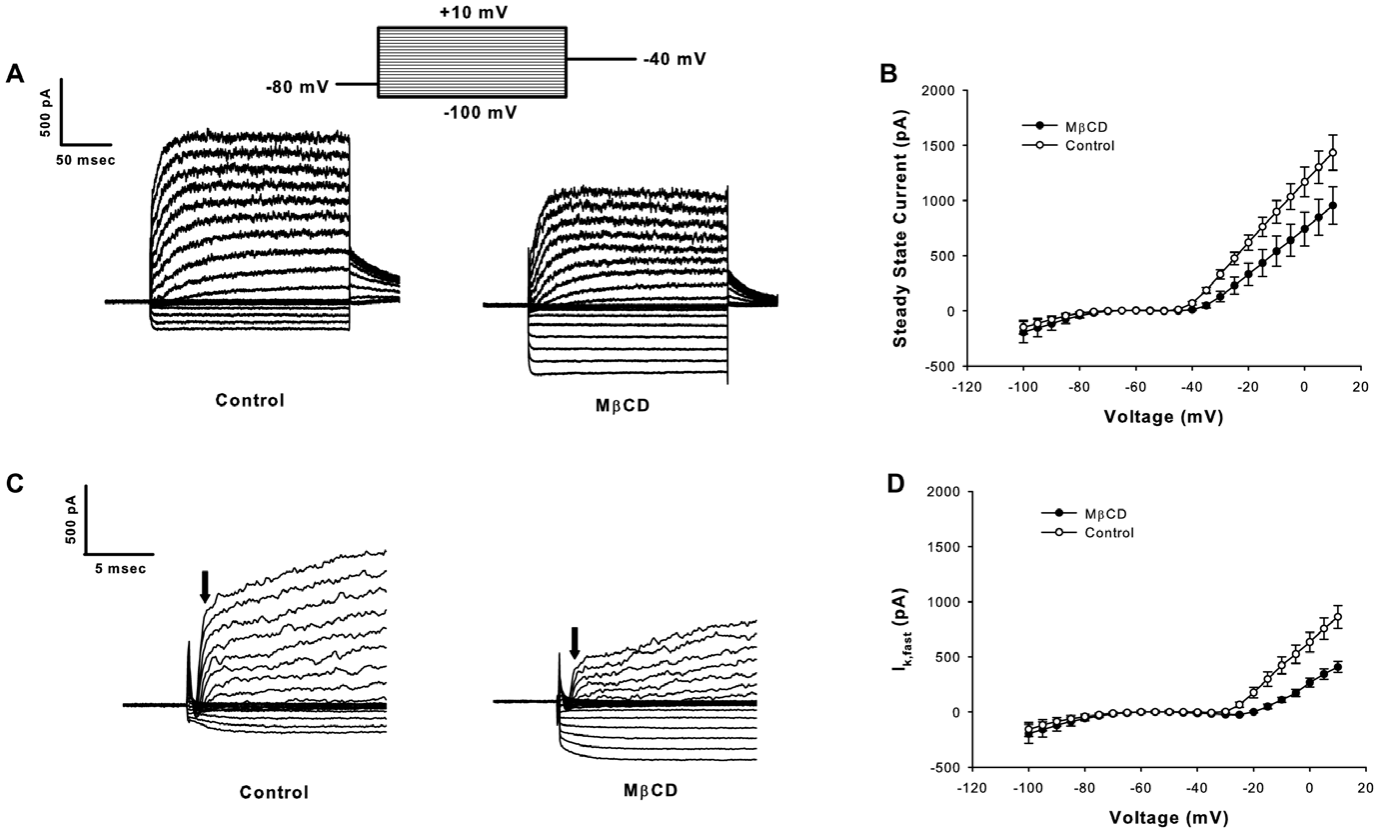
Cholesterol depletion with MβCD reduces fast outward currents in hair cells. (A) Example traces for control and cholesterol-depleted (“MβCD”) reflect the mean drop in outward current following treatment. (B) Steady-state current-voltage curves show reduced outward currents (∼500 pA at 10 mV step) (p<0.001, ANOVA). No difference in inwardly-rectifying potassium currents (Kir) was observed. (C) Examining the fast component of the outward currents (magnified from panel A) illustrates a reduction in the fast component without obvious effects on slow potassium currents. Arrows indicate the current level 1.5 msec following the stimulus (I_K,fast_). (D) I_K,fast_ is reduced following MβCD treatment (∼400 pA at 10 mV step) (p<0.001, ANOVA), while Kir remains unaffected. The reduced slope of the MβCD-treated curve indicates a potential effect on channel biophysics (significant interaction effect, p<0.001, ANOVA).

The overall steady-state outward current was significantly decreased following treatment with MβCD (p<0.001, n = 7−8 cells per treatment group, ANOVA), with a 502pA decrease in the average current at the 10mV voltage step (Figure 2A–B). The total outward potassium current was segregated into fast (I_K,fast_) and slow components for further analysis, where I_K,fast_ was measured 1.5 msec after the voltage step [26]. Pharmacological and molecular studies link I_K,fast_ with the structure and function of BK channel subunits [26], and the remaining component has the characteristics of a slowly-activating voltage-gated delayed rectifier [22]. We investigated the effects of cholesterol depletion on I_K,fast_ as a first step toward understanding the contribution of BK channels to the MβCD effects on total outward K^+^ current. The decrease in I_K,fast_following MβCD treatment accounted for the majority of the decrease in potassium current, with a 402 pA drop in the average I_K,fast_ current at the 10 mV step (Figure 2C–D). MβCD reduced the slope of the I_K,fast_I–V curve in comparison to control cells in the voltage-gated range (>−40 mV), potentially indicating an effect on BK gating characteristics (p<0.001, n = 7−8 per treatment group, ANOVA interaction effect).

**Figure 3:**
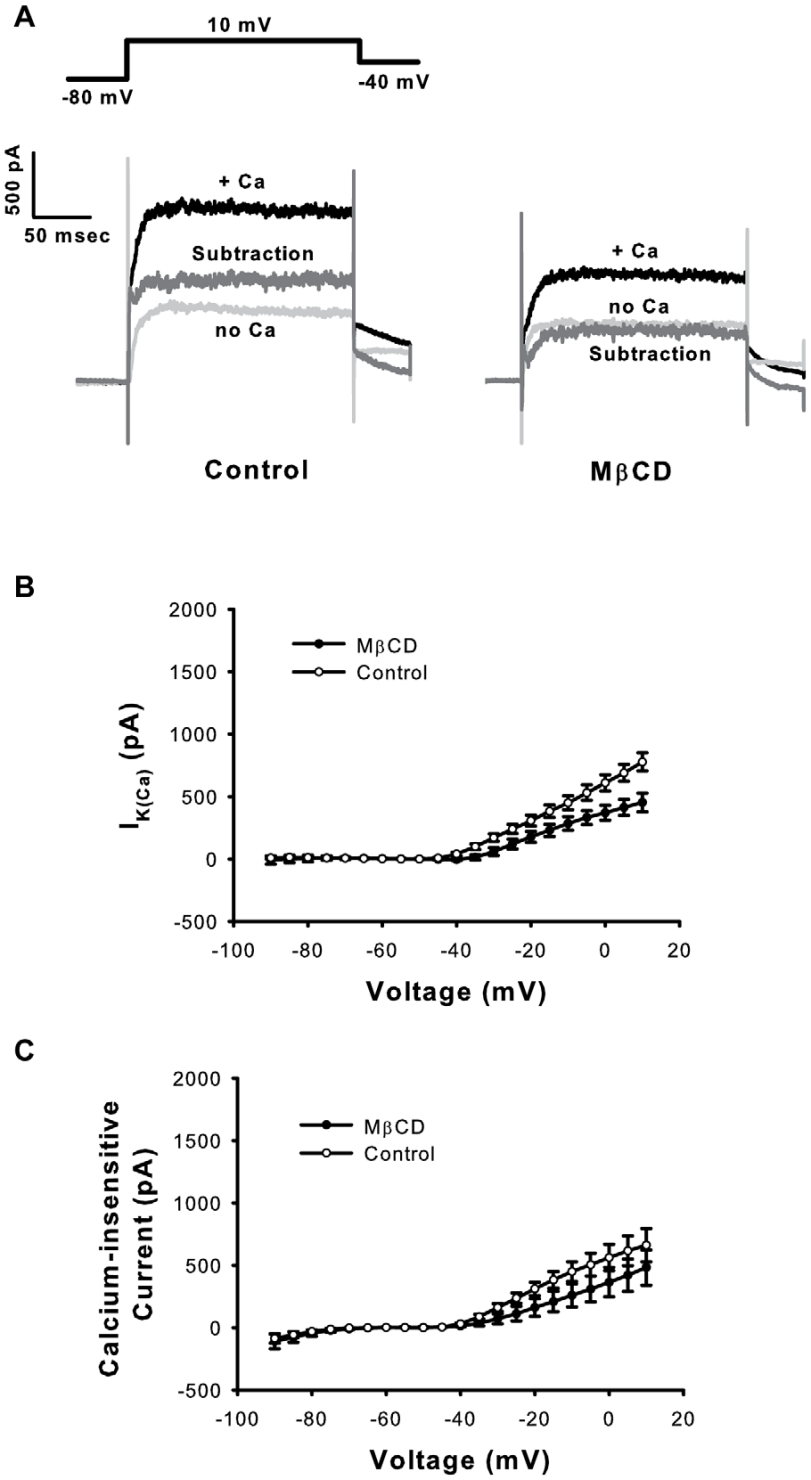
Cholesterol depletion with MβCD reduces calcium-sensitive outward currents (I_K(Ca)_) in hair cells. (A) Representative traces illustrate the reduction of calcium-sensitive outward currents following MβCD treatment. I_K(Ca)_ was defined by subtracting the response to calcium-free from control extracellular solution. (B) Current-voltage (I−V) curves show reduced I_K(Ca)_ following MβCD treatment (p<0.001, ANOVA). (C) Steady-state I−V curves for K^+^ currents recorded in calcium-free external solution show a comparatively lesser reduction following MβCD treatment (p<0.001, ANOVA).

We examined calcium-sensitive and calcium-insensitive currents in control and MβCD-treated hair cells to further explore the relative effects of cholesterol depletion on BK-type and Kv currents[9], [22]. BK dominates the calcium-sensitive outward current in chick tall hair cells (∼90%) [28]. MβCD treatment reduced calcium-sensitive outward currents (I_K(Ca)_) (p<0.001, n = 7−8 cells per treatment group, ANOVA), with a 323 pA drop in the average current at the 10 mV step (Figure 3A–B). Using either fast activation kinetics or calcium sensitivity as the isolation method, BK-type currents were the principal source of the reduction in K^+^ current following MβCD treatment. In the absence of G/Gmax curves constructed from tail currents, we estimated the maximal conductance (Gmax) by fitting a line to the last 5 points of the linear portion of the steady-state calcium-sensitive I-V curve. This analysis method is not definitive as it assumes that the open probability has reached a maximum in this linearly increasing voltage-gated range. Using this estimation method, there was a ∼50% drop in Gmax following MβCD treatment (Control = 16.2 nS, MβCD = 8.3 nS, Figure 3B) (p<0.01, n = 7–8 cells per treatment group, t-test).

### Cholesterol depletion effects in Kv and Kir currents

BK-type currents accounted for the majority of reduced outward current following MβCD-treatment. The remaining reduction was attributable to a smaller, but statistically significant, decrease in voltage-gated, calcium-insensitive currents (p<0.001, Figure 3C, ANOVA). The source of the calcium-insensitive current in chick hair cells is voltage-gated delayed rectifier (Kv) channels [22]. Using the same method of Gmax estimation identified for BK-type current analysis (again, carrying the caveat of assumptions regarding open probability), KvGmax was unaffected by MβCD treatment (Control = 10.4 nS, MβCD = 11.1 nS, Figure 3C) (p = 0.88, n = 7−8 cells per treatment group,t-test). Tail current analysis of calcium-free traces did not reveal significant effects of MβCD on maximum current or half-activation voltage (not shown).

While outward K^+^ currents were significantly decreased by MβCD treatment, there were no significant effects on the inwardly-rectifying K^+^ current in our data set (Kir assessed below −70 mV, Figure 2, ANOVA). MβCD produced the most pronounced effects on the calcium-sensitive, fast outward conductance (BK), without producing a global effect on all K^+^ channels.

### Cholesterol depletion with MβCD increases steady-state calcium currents in hair cells

**Figure 4:**
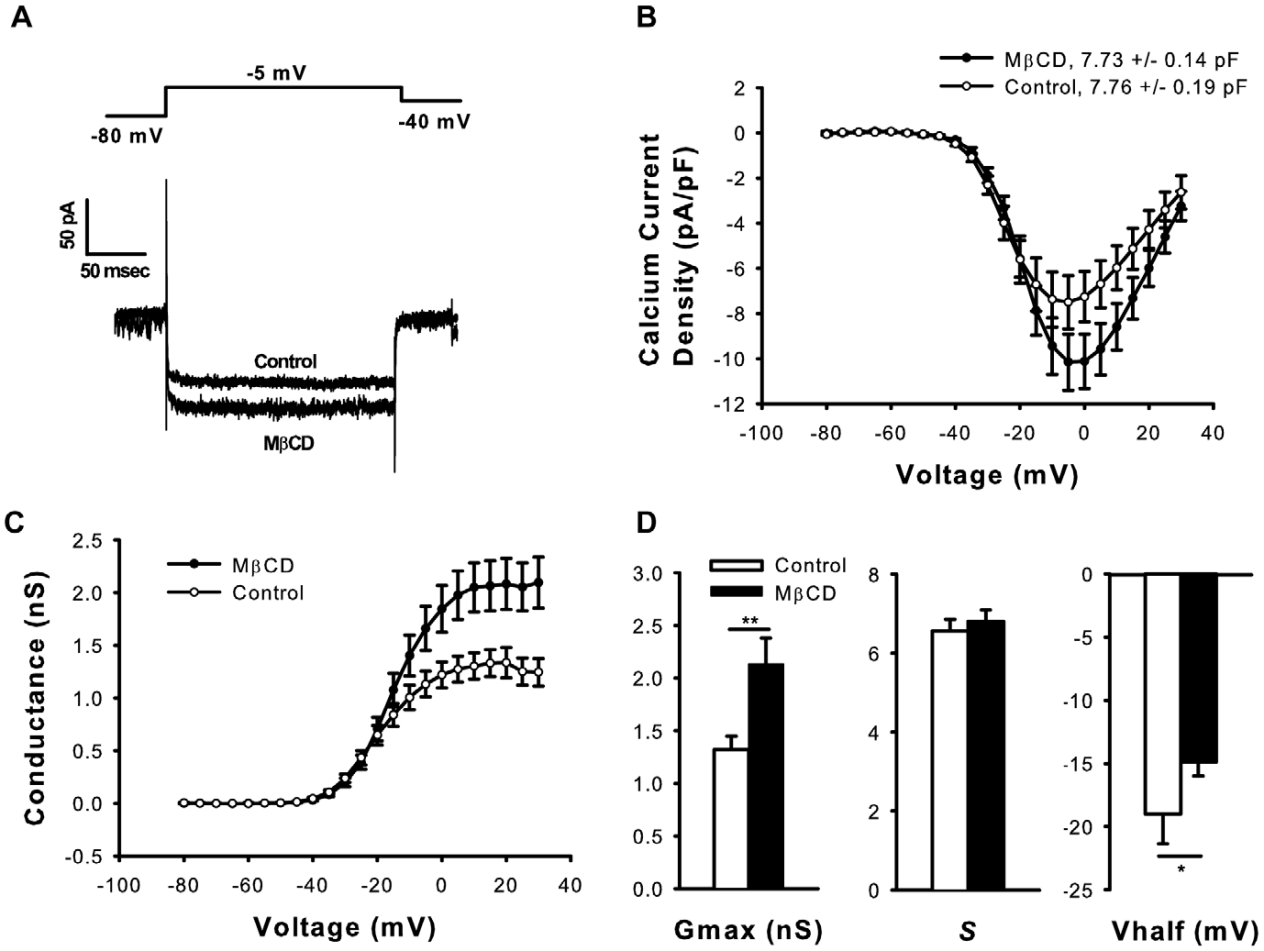
Cholesterol depletion with MβCD increases VGCC currents and maximal conductance. Representative traces (A) and I-V curves (B) demonstrate increased calcium currents following MβCD treatment (p<0.001, ANOVA) without an effect on membrane capacitance. Boltzmann fits to G–V curves (C) demonstrate increased maximal conductance (Gmax) and a rightward shift in Vhalf following MβCD treatment (D). * = p<0.05, *** = p<0.001.

The reduction of I_K(Ca)_ following MβCD treatment could result from an ancillary effect on its calcium source. We studied VGCC function to investigate this possibility. The increase in inward calcium current density (∼30% at peak) following cholesterol depletion ruled out a loss of calcium influx as a cause of reduced calcium-dependent outward current (p<0.001, n = 16 cells per treatment group, ANOVA, Figure 4A–B). The increase in calcium current density was due to increased channel number or activity rather than an effect on membrane capacitance, which was unchanged by treatment (Figure 4B, t-test). Macroscopic conductance (G) was obtained by using Ohm's Law and estimates of the reversal potential and was plotted as a function of voltage (Figure 4C). Single-order Boltzmann fits to G–V curves revealed increased maximum conductance (Gmax, p<0.01, n = 16 cells per treatment group, t-test) and a slight rightward shift in half-activation voltage (Vhalf, p<0.05, n = 16 cells per treatment group, t-test) following MβCD treatment (Figure 4C–D). The data indicate a direct, potentiating effect of cholesterol depletion on VGCC function in auditory hair cells.

### BK is expressed in cholesterol-enriched microdomains

**Figure 5:**
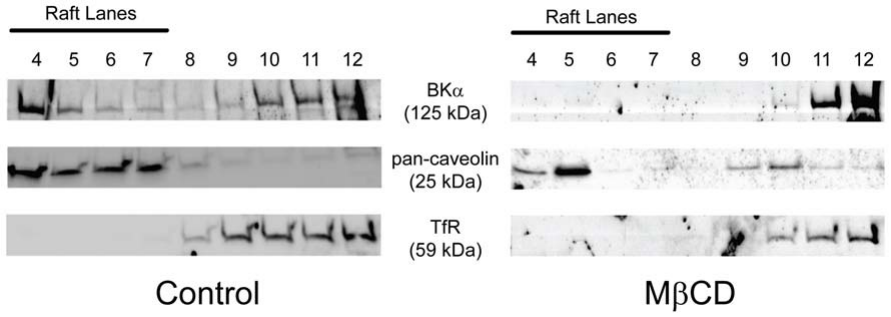
BK channels are found in cholesterol-enriched microdomains. BK expression is found in raft fractions of control cells (lanes 4–7). Caveolin positively identifies raft fractions, and transferrin receptor is expressed in non-raft fractions. MβCD treatment removes BK expression from raft fractions. Representative results from 3 MβCD-treated and 6 control preparations are shown.

An alternative explanation for the change in BK voltage-dependence is that MβCD displaces BK channels from their VGCC calcium source by disrupting cholesterol-dependent microdomains. Lipid rafts are classically defined as sphingolipid- and cholesterol-enriched microdomains which are resistant to disruption with non-ionic detergents [3]. We found BK expression in detergent-resistant membrane fractions, and cholesterol depletion with MβCD shifted BK expression to the soluble fractions (Figure 5, representative result of 3 MβCD and 6 control replicates). Caveolin and transferrin receptor expression were used to confirm the identity of the raft and non-raft fractions, respectively. Caveolin expression was not affected by MβCD treatment. The resistance of caveolin to solubilization with MβCD treatment has been reported elsewhere [29], [30], [31], although caveolin may be removed from raft fractions with higher concentrations of Triton X-100 or with zwitterionic detergents (unpublished observations). Densitometry of control preparations revealed that ∼25% of BK expression was found in lipid raft fractions, and this figure was reduced to ∼5% following MβCD treatment (n = 6 controls, n = 3 MβCD-treated, data not shown). If lipid rafts localize BK channels near VGCCs in the cell membrane, cholesterol depletion could reduce BK function by removing the activating calcium source.

## Discussion

A regulatory role of cholesterol in auditory hair cell physiology is beginning to emerge, with reported roles in outer hair cell (OHC) electromotility and the developmental expression of delayed rectifier channels in chick tall hair cells. Movement of the OHC membrane amplifies the vibrations transmitted to inner hair cells in a frequency-dependent manner, and nonlinear capacitance changes are the hallmark of OHC function [32], [33]. Cholesterol depletion shifts the voltage-dependence of nonlinear capacitance and disrupts the normal punctate pattern of the associated protein ‘motor,’ prestin, when heterologously expressed in HEK293 cells [34]. Interestingly, the cholesterol content of the OHC membrane is developmentally regulated, decreasing with maturation [34]. The developmental influence of cholesterol content extends to delayed rectifier potassium channels in afferently innervated hair cells of the chick [20]. Increased Kv currents following cholesterol depletion eliminated spontaneous action potentials, potentially disrupting the transition to a graded receptor potential [20]. Our data expands cholesterol's known role in the cochlea to include modulation of calcium and BK-type potassium conductances in mature, afferently-innervated hair cells.

Cholesterol's influence on calcium- and calcium-activated potassium channels has potential consequences for auditory hair cell excitability. Cholesterol-dependent alterations in these conductances could re-shape the receptor potential, disturb tuning, affect temporal processing, and change the membrane time constant (and thus, the ability of the cell to respond to repetitive stimuli with high fidelity) [5], [6], [17], [22], [35]. While cholesterol is known to influence Kir and Kv in other cellular systems [2], [36], [37], [38], the inwardly rectifying K^+^ current was unaffected by cholesterol depletion in hair cells in this study, and the impact on Kv was modest. Presumably, the physiological impacts of membrane cholesterol will be greatest in auditory hair cells tuned to higher frequencies, as the density of calcium- and calcium-activated potassium channels increase with frequency and Kir and Kv predominate in low frequency cells [17], [22].

The modulation of VGCC and BK currents by cholesterol, and the associated changes in hair cell excitability,may have implications for sensorineural hearing loss.While the mechanism is unknown and is likely to be multi-faceted, there is a correlation between hearing loss and dyslipidemia[39]. Calcium influx through VGCCs clustered in nanodomains causes exocytosis and neurotransmitter release at the inner hair cell base, and VGCC dysfunction has been linked to deafness in humans [35], [40], [41]. Cholesterol depletion markedly increases the maximal conductance of VGCCs in our data, a fact most likely attributable to increased unitary conductance or channel number. If MβCD increases the number of active VGCCs in the hair cell membrane, a loss of temporal acuity may result, since exocytosis increases linearly with the number of open calcium channels in high-frequency, basal hair cells [5], [35]. In apical cells, where exocytosis increases exponentially with increasing calcium current, phase-locking of low frequency signals could be compromised [5]. Likewise, cholesterol may affect temporal processing via regulation of BK currents. Slowed voltage responses and reduced K^+^ conductance in α-BK knockout mice resulted in variable spike timing of auditory afferents [6]. By modulating VGCC and BK currents, alterations in cochlear cholesterol content may have profound impacts on temporal coding.

Potential mechanisms underlying the impact of MβCD on hair cell currents include direct effects of cholesterol on channel function and co-clustering of VGCC and BK channels in cholesterol-enriched microdomains. Our results show that the effects of cholesterol are channel-specific, where cholesterol depletion augments VGCCs and inhibits BK channels, with absent or less robust effects on Kir and Kv channels respectively. This observation argues against a unifying, global mechanism of MβCD influence on channel function and favors more restricted effects such as disrupting direct cholesterol-channel binding and/or producing localized effects on a heterogeneous lipid environment[2], [38], [42], [43]. Impacts of cholesterol on channel biophysics are supported by significant effects of MβCD on maximal conductance, and the change in I_K,fast_ slope implies a direct effect of MβCD on BK channel number or gating. Disruption of cholesterol-enriched microdomainsmay also contribute to the MβCD effects. As a calcium-activated channel, BK requires close proximity to its calcium source to be efficiently activated upon depolarization, and modeling suggests that calcium-dependent BK channel activation requires VGCCs to be within tens of nanometers of the channel in order to overcome intracellular buffering [44]. The present immunoblotting data and filipin staining are consistent with the localization of BK channels to cholesterol-enriched microdomains at the hair cell base. The I_K,fast_ I-V curve shows a ∼10 mV rightward shift which may be due to either a direct effect on gating or displacement of BK channels from the VGCC calcium source, as decreased intracellular calcium concentration produces a rightward shift in activation [45]. It is possible that the increase in calcium conductance following MβCD treatment partially compensates for displacement of VGCCs and BK channels from their normal co-clustered arrangement at the hair cell base [14], [46].

Membrane cholesterol in the hair cell may have specialized roles at the apical and basal ends of this complex sensory receptor. Our results reveal a new role for cholesterol in the regulation of VGCCs and BK channels which are clustered at the base of mature auditory hair cells. The functional significance of cholesterol at the hair cell apex is unknown, but our observations of ‘floppy’ hair bundles following MβCD treatment indicate a role in the structural stabilization of the rootlet. The rootlet is a filamentous structure that anchors each mechanosensitivestereocilium into the cuticular plate [47]. The details of how and why cholesterol performs this structural role are open to speculation, but the clear implications for mechanotransduction warrant further investigation.

The role of the lipid environment in auditory hair cell physiology is only beginning to be explored. In addition to reported roles in OHC function and delayed rectifier development, our results show that cholesterol influences the VGCC and BK channels necessary for sound encoding. Cholesterol exerts its influence through direct biophysical effects on these channels and may affect the interplay between them via a clustering mechanism. A potential link between the lipid environment and auditory mechanotransduction is a virtual unknown and should be explored. Cholesterol modulation of BK and VGCC channel conductances in hair cells expand our understanding of the mechanisms influencing auditory hair cell excitability and may provide novel pathways for therapies intervening in sensorineural hearing loss.
